# Redox Properties of 3-Iodothyronamine (T1AM) and 3-Iodothyroacetic Acid (TA1)

**DOI:** 10.3390/ijms23052718

**Published:** 2022-02-28

**Authors:** Manuela Gencarelli, Maura Lodovici, Lorenza Bellusci, Laura Raimondi, Annunziatina Laurino

**Affiliations:** 1 Department of Neuroscience, Psychology, Drug Sciences, and Child Health (NEUROFARBA), University of Florence, 50139 Florence, Italy; manuela.gencarelli@unifi.it (M.G.); maura.lodovici@unifi.it (M.L.); laura.raimondi@unifi.it (L.R.); 2 Department of Pharmacology and Physiology, Georgetown University Medical Center, Washington, DC 20007, USA; loghenzpod@gmail.com

**Keywords:** 3-iodothyronamine, 3-iodothyroacetic acid, antioxidant, sirtuin-1, thyroid hormone, lipid peroxidation

## Abstract

3-iodothyronamine (T1AM) and 3-iodothyroacetic acid (TA1) are thyroid-hormone-related compounds endowed with pharmacological activity through mechanisms that remain elusive. Some evidence suggests that they may have redox features. We assessed the chemical activity of T1AM and TA1 at pro-oxidant conditions. Further, in the cell model consisting of brown adipocytes (BAs) differentiated for 6 days in the absence (M cells) or in the presence of 20 nM T1AM (M + T1AM cells), characterized by pro-oxidant metabolism, or TA1 (M + TA1 cells), we investigated the expression/activity levels of pro- and anti-oxidant proteins, including UCP-1, sirtuin-1 (SIRT1), mitochondrial monoamine (MAO-A and MAO-B), semicarbazide-sensitive amine oxidase (SSAO), and reactive oxygen species (ROS)-dependent lipoperoxidation. T1AM and TA1 showed in-vitro antioxidant and superoxide scavenging properties, while only TA1 acted as a hydroxyl radical scavenger. M + T1AM cells showed higher lipoperoxidation levels and reduced SIRT1 expression and activity, similar MAO-A, but higher MAO-B activity in terms of M cells. Instead, the M + TA1 cells exhibited increased levels of SIRT1 protein and activity and significantly lower UCP-1, MAO-A, MAO-B, and SSAO in comparison with the M cells, and did not show signs of lipoperoxidation. Our results suggest that SIRT1 is the mediator of T1AM and TA1 pro-or anti-oxidant effects as a result of ROS intracellular levels, including the hydroxyl radical. Here, we provide evidence indicating that T1AM and TA1 administration impacts on the redox status of a biological system, a feature that indicates the novel mechanism of action of these two thyroid-hormone-related compounds.

## 1. Introduction

Both 3-iodothyronamine (T1AM) and its oxidative metabolite, 3-iodothyroacetic acid (TA1) are endogenous compounds circulating in mammals [1]. Due to the fact that their structure is somehow related to thyroid hormones (THs; i.e., T3 and T4), it was hypothesized they represented end metabolites of THs and possible players of the non-genomic effects of T3. However, their biosynthetic pathway and physio-pathological role remain elusive. Despite this, T1AM and TA1 show pharmacological features in rodents, which suggests the merit to be exploited for their potential clinical effectiveness.

T1AM is considered a multi-target compound [2] and, even more than TA1, its mechanism of action remains mostly elusive and undefined [3]. Overall, the effects of T1AM and TA1, such as their rapid onset, high potency, non-linear dose–effect relationship and signaling, indicate that their mechanisms may include additional or alternative pathways to the recognition of a target. In this respect, some clues suggested that T1AM and TA1 modify the redox status of the system studied.

In fact, at some settings, T1AM’s effects are mediated, at least in part, by the formation of TA1, a product of T1AM oxidative deamination by the mitochondrial monoamine oxidase type B (MAO-B) and/or membrane-bound semicarbazide-sensitive amine oxidases (SSAO). These enzymes produce reactive oxygen species (ROS), which have been found to be casually involved in the pathogenesis of different diseases [4,5,6]. Further, T1AM, but also TA1, protect against excitotoxicity and ischemia-reperfusion injury and ROS-generating conditions, in both in vivo and ex vivo settings [7,8,9,10]. Moreover, Ghanian et al. [11] first reported that T1AM dose-dependently increased the redox ratio of the renal but not cardiac mitochondria of mice. Furthermore, Sacripanti et al. [12] and Assadi-Porter et al. [13] reported that T1AM modulated sirtuin (SIRTs) levels, including sirtuin-1 (SIRT1), a mitochondrial NAD+-dependent deacethylase playing a crucial role in controlling genome stability and considered a target and/or sensor of cell ROS levels [14]. Consistently with this latter, the activation of SIRT1 is a fingerprint signaling of known antioxidants [15,16]. Lastly, in our recent work, [17] we reported that brown adipocytes (BAs) differentiated in the presence of 20 nM T1AM resulted in a cell population (M + T1AM cells) showing a pro-oxidant metabolism compared to cells not exposed to T1AM (M cells).

Drugs acting on the redox status may act as pro- or anti-oxidants or ROS scavengers. These molecules achieve their effects by means of their chemical structure, acting as reducing agents, detoxifying ROS, or “indirectly” preventing ROS formation. This latter class includes compounds controlling the expression/activity of ROS-generating or ROS-detoxifying proteins [18,19]. In this respect, less is known about the antioxidant/pro-oxidant capacity of T1AM and TA1 and whether they can exert control on the expression/activity of the MAO and SSAO, enzymes included in the pharmacokinetics of T1AM and on the antioxidant deacetylase SIRT1.

The aim of this work was to investigate whether T1AM and TA1 were endowed with antioxidant/ROS scavenging activities in in vitro pro-oxidant systems. Next, in the same cell model studied previously, i.e., BAs exposed to 20 nM T1AM or TA1 (M + TA1) or standard medium (M), we investigated the expression level/activity of MAO-A and MAO-B, of the SIRT-1, and of eventual ROS-dependent damages to cell lipids.

## 2. Results

### 2.1. T1AM and TA1 Showed Ferric-Reducing Capacity and Superoxide and Hydroxyl Radical Scavenging Activities

The ferric reducing activity power (FRAP) of T1AM and TA1 was measured as described in the “Methods” section. As positive controls, resveratrol (RESV) and α-tocopherol (α-TOC) were used.

**Figure 1:**
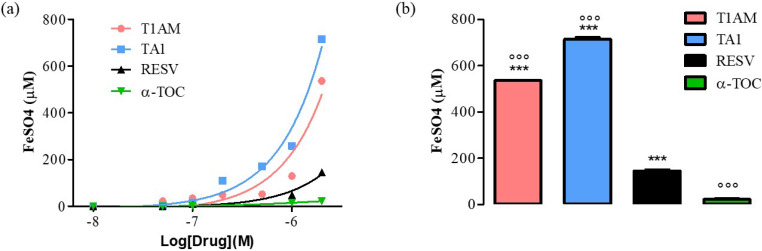
(**a**) Curves produced during the FRAP assay with T1AM, TA1 (10, 50, 100, 200, 500, 1000, 2000 nM), RESV, α-TOC (10, 50, 100, 1000, 2000 nM). (**b**) Bar graph showing FeSO_4_ equivalent (µM) for T1AM, TA1, RESV, and α-TOC at 2000 nM. All experiments were performed in triplicate. Data are expressed as mean ± SEM; °°° *p* < 0.001 vs. RESV, *** *p* < 0.001 vs. α-TOC.

**Figure 2:**
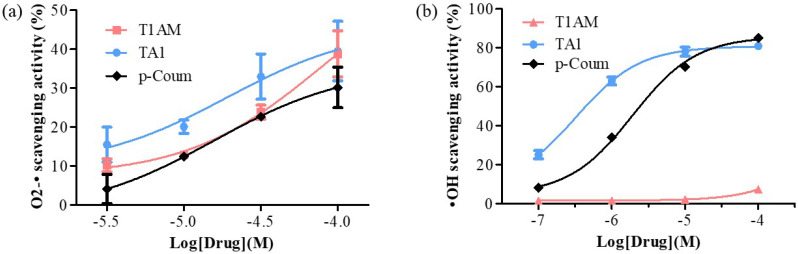
Determination of (**a**) O2-• and (**b**) •OH scavenging activities of T1AM, TA1, and p-Coum (3, 10, 30, and 100 μM and 0.1, 1, 10, and 100 μM, respectively). All experiments were performed in triplicate. Data are expressed as mean ± SEM.

As shown in Figure 1, panel a, the FRAP activity of TA1 and T1AM was evident at concentrations as low as 0.1 μM and it was higher, at 2000 nM (Figure 2, panel b), than that measured for RESV and α-TOC (°°° *p* < 0.001 vs. RESV, *** *p* < 0.001 vs. α-TOC).

T1AM and TA1 also showed free radical scavenging features with some interesting peculiarities. In fact, TA1 showed both superoxide and hydroxyl radical scavenging activities (pEC50 ± SEM of −4.72 ± 0.78 and −6.49 ± 0.10 respectively; Figure 2a,b) with the potency towards the hydroxyl radicals being significantly higher (*** *p* < 0.001) than that measured for the p-coumaric acid (p-Coum) (pEC50 ± SEM −5.71 ± 0.05; Figure 2b).

Instead, our results clearly showed that T1AM had only the capacity to scavenge superoxide radicals (pEC50 ± SEM of −4.12 ± 0.54; Figure 2a) with a potency similar to that of p-Coum (pEC50 −4.80 ± 0.63; Figure 2a).

The FRAP activity of T1AM and of TA1 and their ROS scavenging features indicated that both compounds, at high pro-oxidant conditions, behave as antioxidants.

### 2.2. T1AM and TA1 Modify the Expression and Activity Levels of Pro- and Antioxidant Cell Proteins

We then investigated the expression and activity levels of pro- and antioxidant proteins, including UCP-1, MAO (A and B), SSAO, and SIRT1 in M, M + T1AM, and M + TA1 cells.

#### 2.2.1. Evaluation of UCP-1 Protein and Amine Oxidases mRNA Levels in M + T1AM and M + TA1 Cells

**Figure 3:**
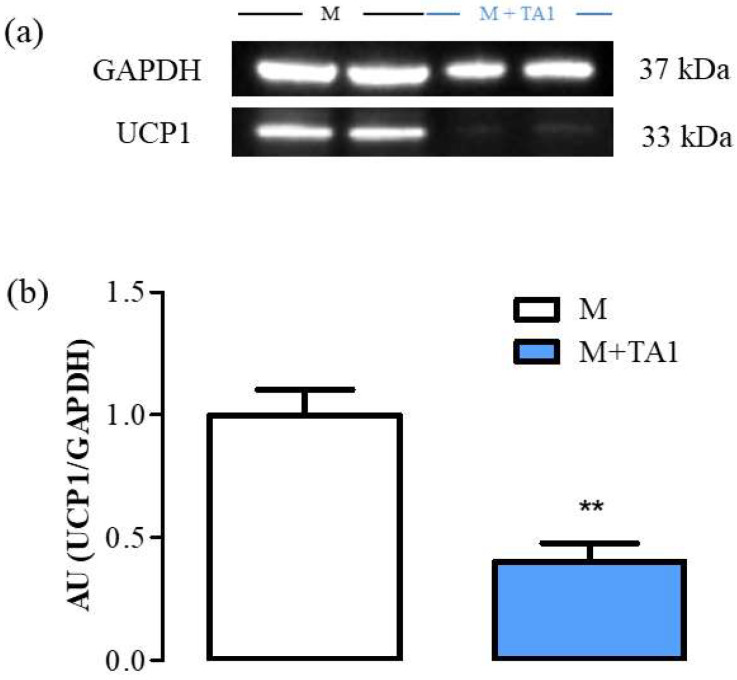
The levels of UCP-1 in M and M + TA1 cells. Cells were obtained as described in the “Methods” section, and analyzed for the expression levels of UCP-1 by Western blot analysis. (**a**) Representative experiment loaded with proteins obtained for two different cell preparations. (**b**) Densitometric analysis is reported as the mean± standard error of the mean (SEM: n = 5 and 6 cell preparations from M and M + TA1, respectively) of arbitrary units (AU; see Methods) ** *p* < 0.01 vs. M cells.

We previously demonstrated that UCP-1 protein levels were similar in M + T1AM and M cells [17]. Instead, in this study, as reported in Figure 3 (panels a and b), the M + TA1 cells showed significantly lower UCP-1 protein expression (Figure 3, panel a, b; ** *p* < 0.01) than the M cells.

#### 2.2.2. Evaluation of mRNA Levels for MAO (A and B) and SSAO in M + T1AM and M + TA1 Cells

**Figure 4:**
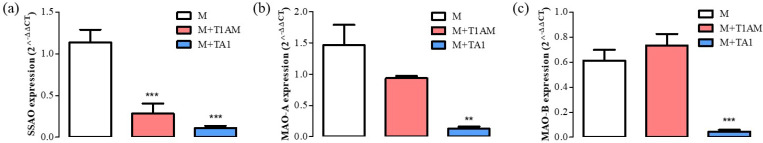
Differentiation marker levels in M, M + TA1, and M + T1AM cells. M, M + TA1, and M + T1AM cells obtained as described in the “Methods” section. The expression levels of mRNA for (**a**) SSAO, (**b**) MAO-A, and (**c**) MAO-B were evaluated by RT-PCR. The mRNA expression of (**a**) SSAO, (**b**) MAO-A, and (**c**) MAO-B were reported as the mean± standard error of the mean (SEM) from four different cell preparations each run in triplicate: ** *p* < 0.01, and *** *p* < 0.001 vs. M cells.

Our results showed that the M + T1AM cells showed lower mRNA levels for SSAO than the M cells, while both cell populations showed similar mRNA levels for MAO-A and MAO-B (Figure 3, panel a, b, ** *p* < 0.01). Instead, the M + TA1 cells presented significantly lower mRNA levels for SSAO, MAO-A, and MAO-B compared to the M cells (Figure 4, panel a, b, c; ** *p* < 0.01; *** *p* < 0.001).

#### 2.2.3. MAO-B Activity

**Figure 5:**
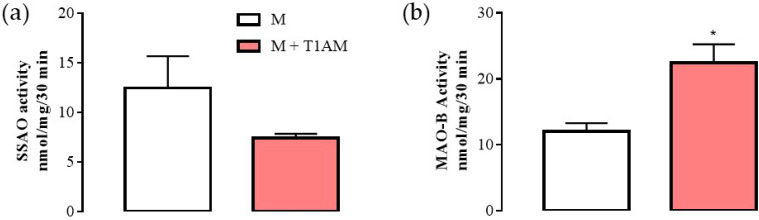
SSAO and MAO-B activities in M and M + T1AM cells. (**a**) SSAO and (**b**) MAO-B activities were measured radiochemically, as described in the “Methods” section. The results are expressed as the means ± SEM of experiments (n = four different cell preparations, each run in triplicate). * *p* < 0.05 vs. M cells.

As shown above, the M + T1AM and M cells expressed mRNA levels for MAO-B at similar levels. However, it is known that the mRNA levels for MAO-B may be not in line with MAO-B enzyme activity [20]. Since MAO-B is the enzyme involved in T1AM degradation producing hydrogen peroxide and then TA1, we decided to evaluate MAO-B enzyme activity by using [^14^]C-benzylamine as substrate at conditions allowing the detection of SSAO or MAO-B respectively. As reported in Figure 5, in M + T1AM cells, SSAO activity (panel a) showed a tendency (not significant) to be lower than in M cells. Conversely, in the same cells, MAO-B activity was found to be significantly higher than in the M cells (panel b, * *p* < 0.05).

#### 2.2.4. SIRT1 Expression and Activity in M + T1AM and M + TA1 Cells

SIRT1 is a NAD+-dependent de-acetylase involved in several cellular processes and functions, a cell redox sensor and among the positive controllers of MAO-A-promoter activity [21]. Therefore, we investigated the expression/activity of SIRT1-1 in our cell populations.

**Figure 6:**
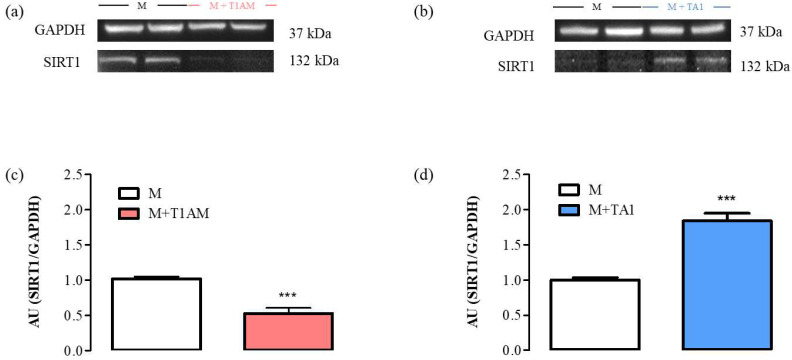
SIRT1 expression levels in M, M + TA1, and M + T1AM cells. M, M + TA1, and M + T1AM cells, obtained as described in the “Methods” section, were analyzed for the expression levels of SIRT1 by Western blot analysis. (**a**,**b**) Representative experiments are shown. Each gel was loaded with proteins obtained from two different cell preparations. (**c**,**d**) Densitometric analysis is reported as the mean± standard error of the mean (SEM: n = four cell preparations) of arbitrary units (AU; see “Methods”. *** *p* < 0.001 vs. M cells.

Our results indicated that the M + T1AM and M + TA1 cells expressed SIRT1 protein levels lower and higher, respectively, than the M cells (Figure 6, Panels a, c; *** *p* < 0.001; Figure 6, Panels b, d; *** *p* < 0.001).

**Figure 7:**
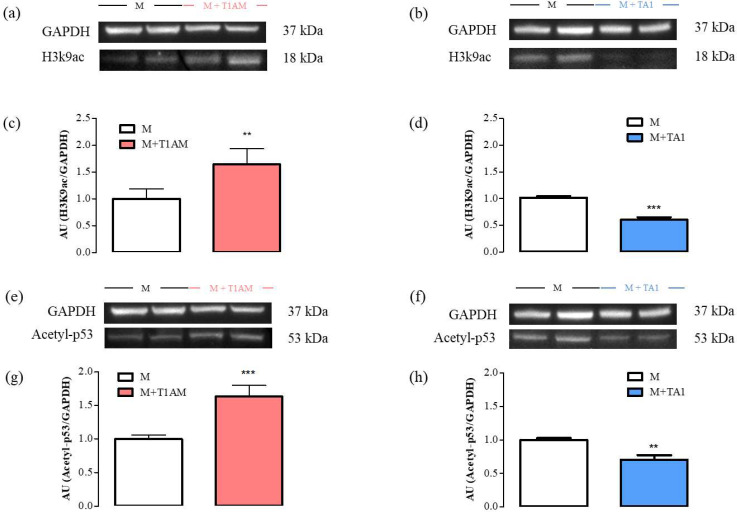
H3K9ac and Acetyl-p53 levels in M, M + TA1, and M + T1AM cells. M, M + TA1, and M + T1AM cells, obtained as described in the Section 4, were analyzed for their levels of H3K9ac and Acetyl-p53 by Western blot analysis, as described in the “Materials and Methods”. In Panels (**a**,**b**,**e**,**f**) representative experiments are shown. Each gel was loaded with proteins obtained for two different cell preparations. (**c**,**d**,**g**,**h**) Densitometric analysis is reported as the mean ± standard error of the mean (SEM: n = 4 cell preparations) of arbitrary units (AU; see “Methods”. ** *p* < 0.01, *** *p* < 0.001 vs. M cells).

The SIRT1 activity was then followed by measuring the expression levels of two of its substrates: H3K9ac and Acetyl-p53. According to our results, the M + T1AM and M + TA1 cells showed higher and lower H3K9ac and Acetyl-p53 protein levels, respectively, than the M cells (Figure 7, panels a, c; ** *p* < 0.01) (Figure 7, panels e, g; *** *p* < 0.001) (Figure 7, panels b, d and panels f, h; *** *p* < 0.001 and ** *p* < 0.01, respectively).

#### 2.2.5. Lipid Peroxidation in M + T1AM and M + TA1 Cells

**Figure 8:**
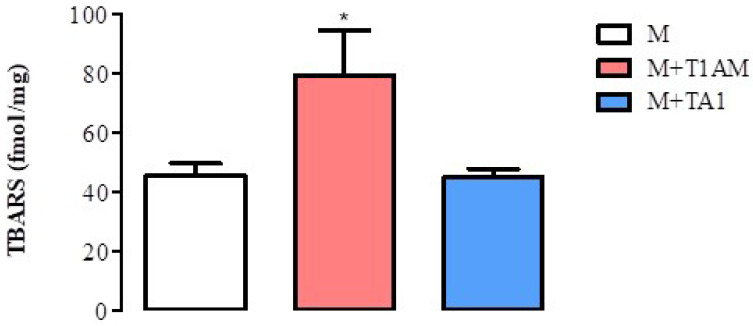
TBARS levels in M, M + TA1, and M + T1AM cells. M, M + TA1, and M + T1AM cells were analyzed for TBARS levels, as described in the “Methods” section. The results are expressed as the mean± standard error of the mean (SEM) from four different cell preparations, each run in triplicate. * *p* < 0.05 vs. M cells.

Since the M + T1AM cells acquired a pro-oxidant metabolism, including a reduction in SIRT1, we decided to verify whether the cells showed signs of ROS-dependent lipid damage, including lipoperoxidation. Consistently with the reduction in SIRT1 levels and activity, M + T1AM cells showed significantly higher TBARS levels than the M cells (Figure 8; * *p* < 0.05). Notably, the TBARS levels were similar in the M and M + TA1 cells.

## 3. Discussion

Here we report, for the first time, that T1AM and TA1, two endogenously occurring thyroid-hormone-related compounds, are endowed with antioxidant/ROS scavenging properties. These features are evident at concentrations ranging from 1 to 100 µM. Consistently, we demonstrate that both compounds can reduce ferric ions with an efficiency greater than that of two known antioxidants, namely RESV and α-TOC. Furthermore, while both molecules are also equipped with scavenging activities in terms of superoxide ions with a potency higher than that of p-coumaric acid, only TA1 is active against hydroxyl radicals, with a potency higher than that of p-coumaric acid.

Reactive oxygen species are produced by basal cell metabolism and have essential signalling roles in differentiation/trans-differentiation processes and aging. Furthermore, ROS are messengers among cell compartments, of cell-to-cell communication, at physiological and pathological conditions. In this respect, terminal adipose differentiation is strictly dependent on ROS cell levels too. Consistently, a reduction in adipocyte maturation and then in obesity is one of the therapeutic indications of the pharmacological supplementation of antioxidants/ROS scavengers [22].

We previously reported that BAs, exposed for 6 days to 20 nM T1AM (M + T1AM cells), acquired pro-oxidant metabolism, including reduced ATP levels and increased basal lipolysis, in the absence of cell toxicity or of any significant reduction in cell differentiation extent. Therefore, we retained this model suitable for extending our study on the mechanistic of T1AM and TA1. In particular, in this study, we investigated the effect of treating BAs with T1AM or TA1 on pro-oxidant cell enzyme expression/activities, i.e., the amine oxidases involved in T1AM pharmacokinetic, and on SIRT1. Further, to confirm the pro-oxidant effects of the M + T1AM cells, we checked whether these cells showed any ROS-dependent damage, including lipoperoxidation.

Our presented results indicate that M + T1AM cells exhibit lipoperoxidation levels higher than those of M cells. This finding clearly indicates that the long-term presence of T1AM induced irreversible ROS-dependent damages to cell lipids, suggesting that drug exposure exhausted the antioxidant defences of cells.

M and M + T1AM cells differ in their MAO-B activity levels. In particular, M + T1AMs have higher MAO-B activity than M cells. MAO-B is the enzyme degrading T1AM, producing hydroxyl radicals that are mostly implicated in lipid peroxidation and not scavenged by T1AM. In addition, the potential higher degradation of T1AM in M + T1AM compared with M cells suggests that the long term-treatment of cells with amine exerts control over the enzymes involved in its own degradation, likely to avoid the accumulation of the amine in cell compartments, an event potentially causing cell death [23,24]. Moreover, the up-regulation of MAO-B activity following T1AM treatment, if confirmed in brain cells, would shed a novel light on the role of MAO-B and T1AM in neurodegenerative diseases, including Parkinson’s disease.

MAO-B activity represents the first step in the enzyme chain, including catalase and aldehyde dehydrogenase (i.e., the MAO cycle) producing TA1 from T1AM. Currently, we do not know how much TA1 is produced in M + T1AM; however, despite this uncertainess, TA1 cannot be excluded as part of the effects described in M + T1AM cells.

The M + TA1 cells presented significantly lower UCP-1 protein levels, mRNA for SSAO, MAO-A and B, early and late markers of adipocyte differentiation [25,26], compared to M cells. These results suggest that TA1 could reduce the extent of adipocyte differentiation, an effect produced by other known antioxidants [27]. Further, consistently with the signalling activated by antioxidants, SIRT1 levels and activity were found to be upregulated in the M + TA1 cells, without any signs of lipid peroxidation. Notably, this is the first evidence indicating that TA1 can also modulate SIRT1 expression/activity and then exert control over genome transcription. Notably, T1AM and TA1 modulate SIRT1 expression/activity in an opposite manner. Since the chemical structure of T1AM and TA1 allows us to exclude the possibility that they can recognize common targets, it is plausible to hypothesize that the modulation of SIRT1 depends on intracellular levels of ROS. In this respect, another important difference between M + T1AM and M + TA1 cells is the expression of MAO-B. In particular, TA1, unlike T1AM, downregulates MAO-B expression in terms of M cells, potentially reducing the intracellular production of hydroxyl radicals, the radical species mostly implicated in BAs differentiation, in the control of SIRT1 and in the lipid oxidation induced by T1AM. In synthesis, at our settings, TA1 behaves as “MAO-B inhibitors” drugs which are known for their protective effects against neurodegeneration and ischemic cell death while working as antidepressants. Consistently, these properties are among the pharmacological effects described so far for TA1 [3,28,29].

Even though our data cannot allow us to conclude on the sequence of the events described, they indicate that the pharmacological activities of T1AM and TA1 include the regulation of the cell redox status, a finding that represents the first mechanism described for TA1. In addition, since the concentrations of both drugs used in this study are close to their endogenous levels, the mechanism described here might also have physio-pathological relevance, including T1AM and TA1 as part of the cell redox balancing systems. In light of these results, the definitive assessment of T1AM and TA1 synthetic pathways assumes increasing importance.

## 4. Materials and Methods

### 4.1. Ferric-Reducing Activity Power (FRAP)

The FRAP activity was measured according to Lodovici et al. [30]. A FRAP reagent solution was freshly prepared by mixing 300 mM acetate buffer, pH 3.6, TPTZ solution (10 mM 2,4,6-tripyridyl-s-triazine (TPTZ) in 40 mM HCl), and 20 mM FeCl_3_·6H_2_O in a volume ratio of 10:1:1. To perform the assay, 0.9 mL of FRAP reagent, 90 µL of distilled water, and 30 µL of T1AM (10–2000 nM) or TA1 (10–2000 nM) were mixed and incubated at 37 °C for 30 min. Resveratrol (RES; 10–2000 nM) and α-tocoferol (α-TOC; 10–2000 nM) were used as positive controls. The absorbance was measured at 595 nm. The antioxidant potential of samples was determined from a standard curve plotted using the FeSO_4_·7H_2_O.

### 4.2. Superoxide Scavenging Activity

The high-throughput superoxide (O2−•) scavenging assay was carried as reported by Tao et al. [31]. Four different concentrations were used for each antioxidant compound in the study. We used p-coumaric acid (p-Coum) as reference compound. The scavenger sample solution (TA1, T1AM, p-Coum, (final concentration range 3–100 μM; 6.67 μL) and 193.33 μL of substrate solution (including 6.43 μL of 4 mM xanthine, 12.86 μL of 4 mM NBT, and 174.04 μL of 0.05 M PBS) were added to each well. Next, 20 μL of 0.04 U/mL XO solution was added to start the reaction. After gentle shaking for 10 s, the reaction mixture was incubated at 37 °C for 20 min. Finally, 20 μL of 0.6 M HCl was added to stop the reaction, followed by shaking for another 10 s. The absorbance at 560 nm was measured. The total volume for each reaction mixture in each well was 240 μL. A blank test without XO and a control test without antioxidants were also conducted. The superoxide scavenging activity was expressed as percentage (%) of superoxide quenching, which was calculated as (1 − B/A) × 100, where B and A are the activities of xanthine oxidase with and without the addition of sample solution, respectively.

### 4.3. Hydroxyl Radicals’ Scavenging Activity

The hydroxyl radicals’ (•OH) scavenging activity was studied using a cell-and-protein-free in vitro system, with the Fenton reaction, as described by Gonzales et al. [32]. The sample solution (T1AM, TA1, p-Coum (0.1–100 μM)) was added to the physiological Ringer’s saline solution at 37 °C, supplemented with the ROS-sensitive fluorescent probe 2′,7′-dichlorodihydrofluorescein (DCF; 5 µM), ferrous iron (Fe_2_SO_4_; 10 µM), and H_2_O_2_ (100 µM) for 30 min. In these conditions, DCF detects hydroxyl radicals but not H_2_O_2_. A blank test without H_2_O_2_ and a control test without antioxidants were also conducted. Fluorescence intensities were measured using a fluorescence microplate reader and excitation/emission filters 485 nm/520 nm. The hydroxyl scavenging activity was expressed as percentage (%) of hydroxyl quenching, which was calculated as (1 − B/A) × 100, where B and A are the activities of Fe^2+^ and H_2_O_2_ with and without the addition of sample solution, respectively.

### 4.4. Cell Culture

The interscapular brown adipose tissue was isolated from one-month-old male Wistar rats (Envigo, San Pietro al Natisone, Italy) according to [17]. For this study, we received permission from the Ethical Committee for Animal Health (176/2017-PR) from the Italian Ministry of Health, and the experimental procedures were carried on in compliance with the European Convention for the Protection of Vertebrate Animals used for Experimental and Other Scientific Purposes (ETS No. 123) and the European Communities Council Directive of 24 November 1986 (86/609/EEC). The authors further attest that all efforts were made to minimize the number of animals used and their suffering. Tissue samples were digested with 2 mg/mL Type-IIa collagenase (Sigma Aldrich, Milano, Italy) prepared in Dulbecco’s Modified Eagle’s Medium (DMEM) High Glucose (GIBCO) at 37 °C for 30 min under agitation. Digested tissue fragments were transferred to a tube containing a doubled volume of digesting solution and DMEM high glucose. The solution was filtered (through Falcon Cell Strainer 70 mesh) and centrifuged twice at 500× *g* for 20 min. Pre-adipocytes were maintained in DMEM high glucose supplemented with 10% FBS (Gibco BRL Life Technology, Grand Island, NY, USA), 1% penicillin-streptomycin solution (100) (BBI Life Science Corporation, Shanghai, China, and 1% L-glutamine (Sigma Aldrich, Milano, Italy) (culture medium) at 37 °C with 5% CO2 atmosphere. Cell differentiation was induced when cells reached 70–80% of confluence adding Differentiation Medium-1 (culture medium supplemented with 10 g/mL insulin, 0.5 mM 1-methyl-3-isobutymethylxanthine, and 1.0 M dexamethasone, all from Sigma-Aldrich, Milan, Italy) for 48 h; thereafter, cells were shifted to Differentiation Medium-2, i.e., culture medium supplemented with 10 g/mL insulin in the absence (M cells) or in the presence of 20 nM of T1AM (M + T1AM cells) or 20 nM TA1 (M + TA1) for 6 days (the medium was changed every 24 h). Each determination was carried out on Day 6. This time point was chosen because it represented the time of complete maturation of BAs [26].

### 4.5. Western Blot

M, M + T1AM, and M + TA1 cells on were lysed on Day 6 in a lysis buffer containing 50 mM Tris HCl (pH = 8), 150 mM NaCl, 1 mM EDTA, 0.1% *w*/*v* SDS, protease, and phosphatase inhibitor cocktail (Thermo Scientific, Monza, Italy). Total protein levels were quantified using the Pierce Protein Assay (Rockford, IL, USA)/BCA (bicinchoninic acid). Twenty micrograms of proteins were separated on 4–20% SDS-PAGE (Thermo Fisher scientific, Waltham, MA, USA) and transferred into PVDF membranes (60 min at 398 mA) using standard procedures. Blots were incubated overnight at 4 °C with specific primary antibody (Table 1) diluted in PBS containing 5% BSA or 5% non-fat dry milk and 0.05% Tween 20. The antigen–antibody complexes were visualized using appropriate secondary antibodies (1:10,000, diluted in PBS containing 5% albumin or 5% non-fat dry milk and 0.05% Tween 20), and left for 1 h at room temperature. Blots were then extensively washed with PBS containing 0.1% Tween 20 and developed using an enhanced chemiluminescence detection system (Pierce, Rodano, Italy). Exposition and developing time were standardized for all blots. Densitometric analysis was performed using the public domain NIH Image program (Image J software Version 1.50i, National Institute of Health, Bethesda, MD, USA). Each gel was loaded with proteins from two different cell preparations. The densitometric analysis presented in the histograms resumed the mean ± SEM of four different cell preparations and was reported as arbitrary units (AU), consisting of the ratio between the level of the target protein expression and that of the GAPDH.

### 4.6. RT-PCR

Total RNA was extracted from M, M + T1AM, and M + TA1 cells on Day 6 using the Macherey-Nagel Nucleo spin RNA (Macherey-Nagel, Dueren, Germany), according to the manufacturer’s protocol. Reverse transcription was performed using the iScript™ Select cDNA Synthesis Kit, Bio-Rad, with 1 μg of total RNA. RT-PCR analyses were performed as described by Laurino et al. [33]. The primers were designed on the basis of the GenBank sequences for Rattus norvegicus (Table 2).

### 4.7. Determination of MAO and SSAO Activities (Radiochemical Assay)

M and M + T1AM cells were lysed in saline phosphate buffer (PBS pH = 7.4), and centrifuged at 1000× *g* × 10 min at 4 °C in order to remove cell debris. Enzyme determination was carried on in the resulting supernatant.

SSAO and MAO-B activity of M and M + T1AM cells were assayed radiochemically using 100 μM (^14^C)-benzylamine (1 μCi/mL; Amersham Biosciences, Amersham, UK) in the absence or in the presence of 1 mM pargyline or 1mM semicarbazide, to inhibit MAO or SSAO activity, respectively. Both inhibitors were pre-incubated for 30 min at 37 °C before the addition of 100 μM (^14^C)-benzylamine.

The labeled substrate was left in contact with enzyme preparations for 30 min at 37 °C. Reactions were stopped by the addition of 20 μL 3 N HCl. The aldehyde produced by enzyme reaction was extracted in ethylacetate (300 μL). The organic phase was separated by brief spinning (1000× *g* × 5 min) and a portion, 150 μL, counted for radioactivity in a β-counter. MAO-B and SSAO activity were then referred to the radioactivity recovered in the organic phase corrected for the radioactivity extracted in the organic phase from a sample where proteins were denatured before the addition of the labeled substrate. Results are expressed as nmol/mg of proteins/30 min.

### 4.8. Thiobarbituric Acid Reactive Substances (TBARS)

Thiobarbituric acid reactive substances (TBARS), markers of lipid peroxidation, were evaluated as previously described by Luceri et al. [34]. M, M + T1AM, and M + TA1 cells on were lysed on Day 6 in 50 µL of PBS. After the addition of 100 µL trichloroacetic acid (TCA), the resulting supernatant (160 µL) was added to 32 µL thiobarbituric acid, 0.12 M (Sigma-Aldrich, Milan, Italy), and heated at 100 °C, for 15 min. The samples were then placed in ice for 10 min and centrifuged at 1600× *g* at 4 °C, for 10 min. The absorbance of the supernatants was measured at 532 nm by using a Wallac 1420 Victor3 Multilabel Counter (Perkin Elmer, Waltham, MA, USA). The amount of TBARS, expressed as fmol/mg of proteins, was calculated using a molar absorption coefficient of 1.56 × 10^−5^ M^−1^ cm^−1^.

### 4.9. Statistical Analysis

Data are expressed as the mean ± SEM of 3–4 independent experiments. Statistical analysis was performed by Student’s *t*-test for paired data or by the one-way ANOVA test followed by the Dunnett multiple comparison test. Before the ANOVA test, a D’Agostino-Pearson omnibus normality test was performed. The threshold of statistical significance was set at *p* < 0.05. Data analysis was performed using the GraphPad Prism 7.0 statistical program (GraphPad software, San Diego, CA, USA).

## Figures and Tables

**Table 1:** List of the primary antibodies used in WB analysis.

Name Product	Dilution	Supplier
Anti-UCP1	1:500 (WB)	Merk-Millipore, Darmstadt, Germany (U6382)
Anti-Histone H3 (acetyl K9 + K14 + K18 + K23 + K27)	1:1000 (WB)	Abcam, Cambridge Biomedical Campus, UK (ab47915)
Anti-SIRT1	1:1000 (WB)	St John’s Laboratory Ltd. London, UK (STJ95667)
Anti-acetyl-p53 Antibody (Lys373, Lys382)	1:1000 (WB)	Merk-Millipore, Darmstadt, Germany (06-758)
Anti-GAPDH	1:2000 (WB)	Cell Signaling Technology, Leiden, The Netherlands (D16H11)

**Table 2:** List of the primers used in RT-PCR.

Gene	Forward Primer 5′ ≥ 3′	Reverse Primer 3′ ≥ 5′	Size (Bp)
MAO-A	GAACCCGAGTCCAAGGATGT	ATGGCCCAAACCATAGGCTG	239
MAO-B	GATGGGCCAAGAGATTCCCAG	CATGGGTCTCCGCAGTTACA	164
SSAO	AATGTGAGCGAGTTGGTGGT	TGTGTCCTGGCGTTTGTAG	211
18s	AAACGGCTACCACATCCAAG	CCTCCAATGGATCCTCGTTA	155

## Data Availability

The data presented in this study are available on request from the corresponding author.
